# Age-Influenced Receptors of Advanced Glycation End Product Overexpression Associated With Osteogenic Differentiation Impairment in Patients With Type 2 Diabetes

**DOI:** 10.3389/fendo.2021.726182

**Published:** 2021-08-26

**Authors:** Mattabhorn Phimphilai, Peraphan Pothacharoen, Prachya Kongtawelert

**Affiliations:** ^1^Division of Endocrinology, Department of Internal Medicine, Faculty of Medicine, Chiang Mai University, Chiang Mai, Thailand; ^2^Thailand Excellence Center for Tissue Engineering and Stem Cells, Department of Biochemistry, Faculty of Medicine, Chiang Mai University, Chiang Mai, Thailand

**Keywords:** advanced glycation end products, receptor of advanced glycation end products, osteogenic differentiation, peripheral blood derived mononuclear cells, type 2 diabetes

## Abstract

Preclinical studies have found impaired osteogenic differentiation to be associated with type 2 diabetes (T2DM), which is related to skeletal accumulation of advanced glycation end products (AGEs). Our previous study also showed impaired osteogenic differentiation in peripheral blood-derived mononuclear cells (PBMC) isolated from patients with long-standing T2DM, which is conceivably due to the overexpression of receptor of advance glycation end products (RAGE) and the enhancement of cellular apoptosis. However, the existence of RAGE overexpression in earlier stages of diabetes remains unclear, as do the factors influencing that RAGE overexpression. This cross-sectional study enrolled 40 patients with T2DM treated with metformin monotherapy and 30 age-matched non-diabetic controls (NDM) to investigate the overexpression of RAGE in PBMC derived from patients with earlier stage diabetes, as well as to explore its determining factors. Almost all (90%) PBMC-isolated from NDM (NDM-pD) expressed osteoblast-specific genes including *ALPL*, *BGLAP*, *COL1A1*, and *RUNX2/PPAR* while only 40% of PBMC-derived from diabetic patients (DM-pD) expressed those genes. By using age- and pentosidine-matched NDM-pD as a reference, *AGER* and *BAX/BCL2* expression in PBMC isolated from diabetic patients showing impaired osteoblast-specific gene expression (DM-iD) were 6.6 and 5 folds higher than the reference while *AGER* and *BAX/BCL2* expression in DM-pD were comparable to the reference. *AGER* expression showed a significant positive correlation with age (r=0.470, *p*=0.003). The multivariate analysis demonstrated that both age and *AGER* expression correlated with the potential for osteogenic differentiation in the PBMC isolated from patients with diabetes. In conclusion, this study showed osteogenic differentiation impairment in approximately half of PBMC derived from type 2 diabetic patients receiving metformin monotherapy. Both *AGER* and *BAX/BCL2* overexpression were demonstrated only in PBMC-isolated from diabetic patients with poor osteogenic differentiation. Therefore, this study not only illustrated the existence of RAGE overexpression in PBMC derived from patients with early stages of T2DM but also strengthened the linkage between that RAGE overexpression and the retardation of osteogenic differentiation. Age was also shown to be a positive influencing factor for RAGE overexpression. Furthermore, both age and RAGE overexpression were demonstrated as independent risk factors for determining osteogenic differentiation potential of the PBMC-isolated from T2DM.

## Introduction

Type 2 diabetes (T2DM) is a major health issue worldwide. It is a metabolic disorder characterized by insulin resistance and chronic hyperglycemia, contributing to multiple devastating microvascular and macrovascular complications. It is well documented that chronic hyperglycemia accelerates the accumulation of advanced glycation end products (AGEs) (1–3). These are non-enzymatic modifications of proteins which usually slowly accumulate in long-lived substrates in aging animals (4) and humans (5, 6). The accumulation of AGEs is one of the main mechanisms linking hyperglycemia and the chronic microvascular and macrovascular complications which develop in cases of diabetes (7–9).

T2DM is associated with a decrease in bone turnover (10, 11), changes in bone microarchitecture (12) and increases in the risk of fragility fractures (13–18) with a preserved bone mineral density (15, 16, 18). These indicate an adverse effect of diabetes on bone quality. Furthermore, the risk of fragility fractures increases with poorer glycemic control (19–21), suggesting an impact of hyperglycemia on fragility fractures, the most serious complication of osteoporosis. The skeletal accumulation of AGEs, which is accelerated in the presence of hyperglycemia, may contribute to those phenomena in diabetic patients. The non-enzymatic glycation of type 1 collagen of the bone competes with the enzymatic collagen crosslinking, yielding a skeletal accumulation of AGEs such as pentosidine. Multiple preclinical studies have demonstrated that the accumulation of AGEs altered the mechanical properties of bone (22–24) and interferes with the functions of bone cells including osteoblasts (25–30). In clinical studies Furst and colleagues (31) demonstrated an inverse correlation between skin AGE accumulation and bone material strength in patients with T2DM. Furthermore, a positive correlation has been shown between serum pentosidine and vertebral fractures (32–34). In contrast, an endogenous secretory receptor of AGE (esRAGE), a neutralizing molecule of AGE, showed a negative correlation with vertebral fractures in T2DM (34, 35).

AGEs act *via* binding to their specific receptor, the receptor of AGE (RAGE). Following binding with AGEs, RAGEs initiate multiple signal cascades including inflammatory and apoptotic pathways. Several *in vitro* studies in osteoblast lineage cells have demonstrated that AGEs-dependent RAGE activation inhibited osteoblast differentiation (36–39) and enhanced osteoblast apoptosis (25, 28, 39, 40). In our previous study in human subjects with T2DM, impaired osteogenic differentiation and enhanced cellular apoptotic signals were demonstrated, both of which are possibly linked to cellular RAGE overexpression in individuals with T2DM (41).

Our previous study illustrated higher *AGER* expression in peripheral-blood derived mononuclear cells (PBMC)-isolated from patients with long-standing T2DM compared to those in a matched non-diabetic control group, suggesting higher cellular RAGE activation in individuals with long-standing T2DM (41). Moreover, the cellular RAGE overexpression was positively associated with enhanced cellular apoptotic signals and then was potentially involved in the impairment of osteogenic differentiation potential of the cells (41). However, the existence of cellular RAGE overexpression in earlier stages of diabetes remains to be elucidated, as do the factors influencing that RAGE overexpression. It is known that the peripheral blood-derived mesenchymal stem cells can differentiate into multiple cell types, including adipocytes, chondrocytes and osteoblasts (42–44). Our previous study also showed the osteogenic differentiation potential of the PBMC-isolated from both healthy volunteers and patients with T2DM (41). To determine osteogenic differentiation of stem cells using the least invasive measures, this study was conducted using PBMC-derived from participants to investigate: 1) the osteogenic differentiation potential of PBMC-isolated from T2DM patients receiving metformin monotherapy, 2) the existence of cellular RAGE overexpression and the effects of that RAGE overexpression on osteogenic differentiation and cellular apoptotic signals, and 3) factors influencing that cellular RAGE overexpression.

## Materials and Methods

### Ethical Statements

This study was a cross-sectional study, performed at Maharaj Nakorn Chiang Mai Hospital, Chiang Mai University, Chiang Mai, Thailand. The study was approved by the Research Ethics Committee of the Faculty of Medicine, Chiang Mai University (MED-2557-02609). All participants signed an informed consent agreement before they were enrolled on the study.

### Study Population and Sample Collection

Metformin is the first-line medication recommended for treatment of T2DM by the majority of international guidelines. In Thailand, metformin monotherapy is mostly used as initial therapy for patients with T2DM. Therefore, in this study, T2DM patients taking metformin monotherapy were enrolled to be representative of early-stage diabetes. Age-matched non-diabetic individuals were enrolled as a control group. The exclusion criteria were as follows: females with serum creatinine higher than 1.4 mg/dL or males with serum creatinine above 1.5 mg/dL; individuals who use thiazolidinedione, steroids, immunosuppressive agents, anti-resorptive agents or anabolic agents for osteoporosis, and individuals with metastatic or hematologic malignancy. Venous blood (35-40 mL) was collected from all enrolled participants to isolate the PBMC, and to determine serum levels of pentosidine (Elabscience Biotechnology, WuHan, Hubei, China) and sRAGE (R&D, Minneapolis, MN, USA). Fasting plasma glucose (FPG), glycated hemoglobin (HbA1c), low-density lipoprotein cholesterol (LDL-C) and serum creatinine were assessed using standardized procedures at a central laboratory of the Faculty of Medicine, Chiang Mai University. Glomerular filtration rate (eGFR) was calculated using the Chronic Kidney Disease Epidemiology Collaboration (CKD-EPI) method. Fracture risk estimation was estimated from The Fracture Risk Assessment Tool (FRAX^®^) using the Thailand database (45).

### Isolation and Culture of Human Peripheral Blood-Derived Mononuclear Cells (PBMC)

PBMC were isolated from the 35-40 mL of peripheral venous blood using density gradient centrifugation as described in our previous study (41). In brief, the venous blood was centrifuged at 1500 rpm for 5 minutes. After plasma was removed, the remaining fraction was first diluted with an equal volume of DMEM (Gibco, Grand Islands, NY, USA) and then overlaid on Histopaque (specific gravity 1.077 g/mL; Sigma-Aldrich, St Louis, MO, USA) and finally centrifuged at 4000 rpm for 30 minutes. The PBMC were isolated from the mononuclear cell layer and plated in 24-well culture plates. These were then cultured in RPMI supplemented with 10% (v/v) fetal bovine serum (Gibco, Grand Islands, NY, USA). After removing the floating cells, the plastic-adhered cells were cultured in DMEM supplemented with 10% (v/v) fetal bovine serum (Gibco, Grand Islands, NY, USA), called non-osteogenic-inducing medium in the present study, for 7-10 days until confluence. To induce osteogenic differentiation, the plastic-adhered cells were cultured in non-osteogenic-inducing medium until reaching 50% confluence. They were then changed to an osteogenic-inducing medium (DMEM supplemented with 10^-7^ M dexamethasone, 60 μM ascorbic acid and 10 mM β-glycerophosphate) and cultured for a further 21 days.

### Analysis for the Expression of Osteoblast-Specific Genes, *AGER*, and Cellular Apoptotic-Associated Genes

To examine gene expression total RNA was extracted using the illutraRNA spin Mini Kit (GE Healthcare Life Science, Buckinghamshire, UK) in accordance with the manufacturer’s instructions. Total RNA (500 ng) of each sample was used for reverse transcription into cDNA using an iScript™cDNA Synthesis Kit (Bio-Rad, Hercules, CA, USA) in accordance with the manufacturer’s protocol. Afterwards, the cDNA was analyzed by real-time quantitative polymerase chain reaction (real-time qPCR)(SsoFast EvaGreen Supremixes; Bio-Rad, Hercules, CA, USA). The reaction took place at 45 cycles of 5 seconds at 95**°**C, 10 seconds at 60**°**C and 30 seconds at 72**°**C using the Applied Biosystems 7500/7500 Fast Real-Time PCR system. The total RNA extracted from the PBMC, cultured both in non-osteogenic and osteogenic-inducing media, was used to determine: 1) osteoblast-specific genes including *ALPL, BGLAP, COL1A1* and *RUNX2* for representing osteoblast differentiation and 2) *PPAR-γ* which is a transcription factor driving towards adipocytes for evaluating signals against osteoblast differentiation. Differentiation towards osteoblasts was defined by the increment of expression of all osteoblast-specific marker genes including *ALPL*, *BGLAP* and *COL1A1*, as well as the increment of the *RUNX2/PPARγ* ratio. In contrast, the total RNA extracted only from the PBMC cultured in the non-osteogenic-inducing medium was used to determine: 1) *AGER* expression to elucidate cellular RAGE overexpression, and 2) *BAX* and *BCL2* expression for the evaluation of cellular apoptotic signals. The primers were purchased from Invitrogen (**Table 1**). In the real-time qPCR, the *GAPDH* gene was used for normalization of the relative expression levels for each primer set by the 2^(-ΔΔCT)^ method.

**Table 1:** Sequences of real-time qPCR primers.

Genes	Primer sequence (5’-3’)	
	Forward	Reverse
*ALPL*	CATGGCTTTGGGCAGAAGGA	CTAGCCCCAAAAAGAGTTGCAA
*AGER*	GCTGGAATGGAAACTGAACACAGG	TTCCCAGGAATCTGGTAGACACG
*BAX*	TGGAGCTGCAGAGGATGATTG	GAAGTTGCCGTCAGAAAACATG
*BCL2*	CATGCTGGGGCCGTACAG	GAA CCGGCACCTGCACAC
*BGLAP*	GAAGCCCAGCGGTGCA	CACTACCTCGCTGCCCTCC
*COL1A1*	CAGCCGCTTCACCTACAGC	TTTTGTATTCAATCACTGTCTTGCC
*GAPDH*	CCCTTCATTGACCTCAACTA	AGATGATGACCCTTTTGGCT
*PPARγ*	AAAGAAGCCAACACTAAACC	CTTCCATTACGGAGAGATCC
*RUNX2*	TCTTAGAACAAATTCTGCCCTTT	TGCTTTGGTCTTGAAATCACA

### Statistical Analysis

All descriptive data are reported as mean ± standard deviation. An independent *t*-test was used to compare all continuous parameters while a Chi square test was used to compare binary parameters. Linear regression analysis was used to demonstrate factors correlating with osteoblast differentiation. Pearson’s correlation was used to identify the correlation between parameters, the exception being the correlation between *AGER* expression and other parameters which was done using Spearman’s correlation. A *p*-value of less than 0.05 was used as a measure of statistical significance. All statistical analyses were done with SPSS version 23.0.

## Results

### Demographic Data and Clinical Characters of Study Participants

The study included 40 patients with T2DM treated with metformin monotherapy (DM) and 30 age-matched participants without diabetes (NDM). All patients with T2DM were diagnosed as DM using the FPG criteria with a cut-off value of 126 mg/dL as recommended by the American Diabetes Association. Age, gender, body mass index (BMI), systolic blood pressure (SBP), diastolic blood pressure (DBP), LDL-C, eGFR, and 10-year fracture risk as determined by FRAX^®^ were comparable in both NDM and DM groups (**Table 2**). With comparable levels of blood pressure in both DM and NDM groups, the usage rates of anti-hypertensive agents, including angiotensin-converting enzyme inhibitors (ACEI), angiotensin II receptor blockers (ARB), dihydropyridine calcium channel blockers (DHP-CCB) and thiazide-like diuretics, were higher in DM group than in NDM group. With comparable levels of LDL-C in both groups, the rate of statin use was higher in DM group than in NDM group. However, the difference in anti-hypertensive drugs and statins usage rates did not reach statistical significance (**Table 2**). Metformin was not applicable to NDM group. In the group with diabetes, the duration of diabetes was 5.5 ± 4.1 years with a 47.5% prevalence of microvascular complications and 10% prevalence of macrovascular complications. Metformin was the only anti-hyperglycemic agent used in all diabetic participants with a dosage of 1652.5 ± 627.4 mg/day. Both FPG and HbA1c levels were significantly higher in the DM group than those in NDM (**Table 2**). Serum pentosidine was significantly elevated in DM compared to that in NDM (6.1 ± 3.6 ng/mL **vs** 4.0 ± 2.1 ng/mL, *p*=0.03), suggesting accelerated AGEs accumulation in T2DM (**Table 2**). In contrast to pentosidine, serum sRAGE, a decoy receptor of AGEs, was comparable in both DM and NDM groups (527.1 ± 249.7 pg/mL **vs** 599.4 ± 422.1 pg/mL, *p*=0.374). Even though FPG, HbA1c and serum pentosidine levels were significantly higher in DM than in NDM group, serum pentosidine level showed no correlation with either FPG (*r=*0.065, *p* =0.598) or HbA1c (*r*=0.062, *p*=0.668). In addition, serum pentosidine level showed no correlation with other parameters, including age, BMI, eGFR and sRAGE level. Fracture risk as estimated by FRAX using the Thailand database was comparable between DM and NDM groups (**Table 2**).

**Table 2:** Clinical characteristics of the study participants.

Parameter	NDM (n = 30)	DM (n = 40)	*p*-value
**Demographic data**			
Age (years)	59.7 ± 7.7	58.1 ± 6.8	0.355
Gender (% female)	63.3	60.0	0.487
BMI (kg/m²)	24.6 ± 3.9	25.8 ± 4.3	0.269
SBP (mmHg)	130.8 ± 13.1	129.1 ± 11.8	0.553
DBP (mmHg)	78.9 ± 9.8	75.5 ± 9.4	0.150
FPG (mg/dL)	95.8 ± 9.6	138.6 ± 26.0	<0.0001
HbA1c (%)	5.9 ± 0.50	7.5 ± 0.9	<0.0001
LDL-C* (mg/dL)	111.4 ± 31.3	100.5 ± 42.4	0.222
DM duration (years)	–	5.5 ± 4.1	–
Metformin dosage (mg/day)	–	1652.5 ± 627.4	–
Other drugs (% use)			
•ACEI or ARB** •DHP-CCB^#^ •Thiazide-like diuretic •Statins	44.8 51.7 13.8 66.7	67.5 57.5 20.0 79.5	0.084 0.807 0.749 0.275
Microvascular complications (%)	–	47.5	–
eGFR (ml/min)	86.8 ± 14.3	85.5 ± 17.0	0.745
Macrovascular complications (%)	–	10.0	–
FRAX: 10-year risk of hip fractures (%)	0.7 ± 0.9	0.5 ± 0.9	0.399
FRAX: 10-year risk of osteoporotic fractures (%)	3.4 ± 2.0	2.7 ± 1.8	0.166
**Serum markers**			
Pentosidine (ng/mL)	4.0 ± 2.1	6.1 ± 3.6	0.030
Soluble RAGE (pg/mL)	599.4 ± 422.1	527.1 ± 249.7	0.374

*LDL-C, low-density lipoprotein cholesterol; **ACEI, angiotensin-converting enzyme inhibitors; **ARB, angiotensin II receptor blockers; ^#^DHP-CCB, dihydropyridine calcium channel blockers.

### Age Was an Independent Risk Factor for Preserving Osteogenic Differentiation Potential in Type 2 Diabetes

**Figure 1:**
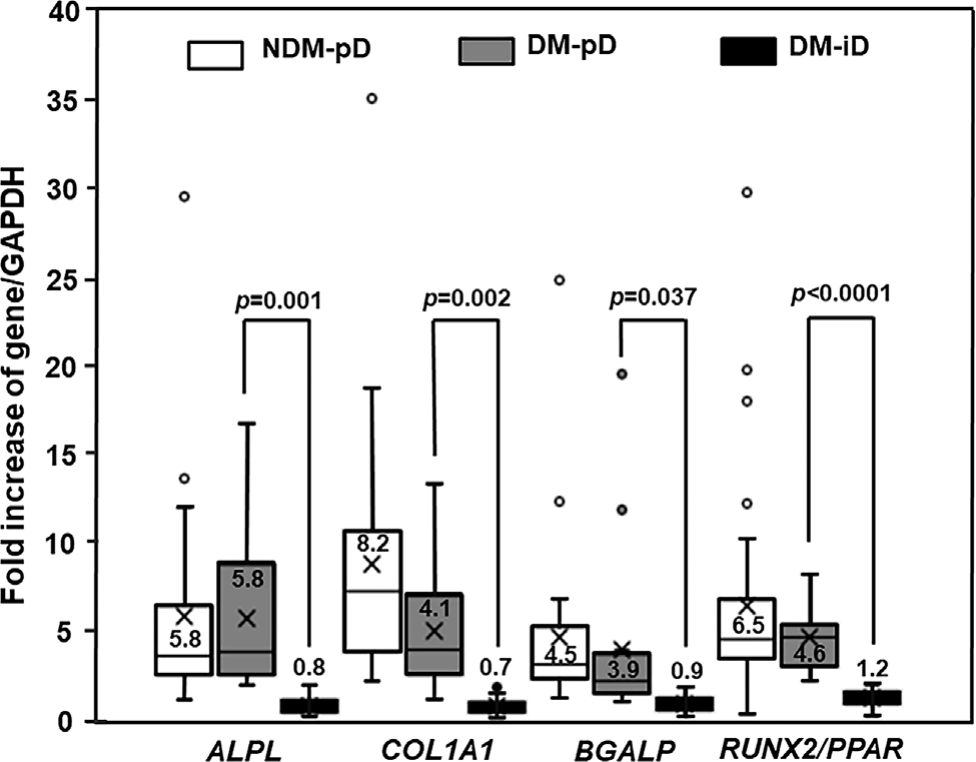
Osteogenic differentiation marker expression. Box and whisker plots to show comparison of osteoblast-specific gene expression in participants without diabetes showing preserved osteogenic differentiation potential (NDM-pD), patients with diabetes showing preserved osteogenic differentiation potential (DM-pD) and patients with diabetes showing impaired osteogenic differentiation potential (DM-iD)(mean ± SD). DM-pD had higher levels of expression of ALPL, COL1A1, BGLAP and RUNX2/PPARγ ratio (RUNX2/PPAR) than those in DM-iD by 7.3, 5.9, 4.3 and 3.8 times, respectively. DM-pD had significantly higher levels of expression of all osteoblast-specific genes than those in DM-iD but had comparable levels of expression of all osteoblast-specific genes to those in NMD-pD.

The PBMC-derived from diabetic patients showed a reduced potential to differentiate towards osteoblasts compared with those from non-diabetic controls. By using osteoblast-specific gene expression as a marker for osteogenic differentiation potential, the isolated PBMC from DM were divided into 2 groups including DM with preserved osteogenic differentiation potential (DM-pD) and DM with impaired osteogenic differentiation potential (DM-iD). Forty percent (16/40) of the PBMC-isolated from diabetic patients showed expression of osteoblast-specific genes including *ALPL*, *BGLAP*, *COL1A1* and *RUNX2/PPAR*; therefore, the isolated-PBMC in this group were classified as DM-pD. The other 60% (24/40) of PBMC-isolated from DM did not express those osteoblast-specific genes, so the isolated-PBMC in this group were classified as DM-iD. While almost all the isolated PBMC from NDM expressed osteoblast-specific genes (NDM-pD), only 40% of the isolated PBMC from DM expressed osteoblast-specific genes (DM-pD) (90% *vs* 40%; *p*<0.0001). As shown by the *RUNX2/PPAR* ratio, the PBMC-derived from DM-pD expressed *RUNX2*, a master transcription factor for osteogenic differentiation, and *PPARγ*, a transcription factor driving differentiation against osteoblasts, at a similar extent to that of NDM-pD but at 3.8 times higher than that of DM-iD (**Figure 1**). Moreover, the PBMC-isolated from DM-pD showed the expression of *ALPL*, *COL1A1* and *BGLAP* to be comparable to those of NDM-pD but at 7.3, 5.9, and 4.3 times higher than those of DM-iD (**Figure 1**). Factors determining osteoblast differentiation were analyzed in both NDM and DM groups. The multivariate analysis demonstrated that being diabetic is the only factor determining differentiation towards osteoblasts. Being diabetic increased the risk of osteogenic differentiation impairment 13.5 times (OR 13.5; 95% CI 3.21-77.91; *p*<0.001).

Forty percent of the PBMC-isolated from diabetic patients demonstrated a similar level of osteoblast-specific gene expression compared to those from non-diabetic individuals, indicating the preservation of osteogenic differentiation potential only in some individuals with type 2 diabetes. We next determined the factors influencing the preservation of osteogenic differentiation potential in the PBMC-derived from diabetic patients. Ten percent of PBMC-isolated from non-diabetic controls (3/30) which did not express osteoblast-specific genes, was excluded from the following analysis. When comparing DM-pD and NDM-pD, patients in the DM-pD group had significantly higher FPG (137.7 ± 30.2 mg/dL *vs* 96.1 ± 9.4 mg/dL, *p*<0.0001), HbA1c (7.5 ± 1.1% *vs* 6.0 ± 0.4%, *p*<0.0001) and serum pentosidine (7.3 ± 4.7 ng/mL *vs* 3.7 ± 1.8 ng/mL, *p*=0.008) than those in the NDM-pD group (**Table 3**). In addition, individuals with DM-pD were younger (54.4 ± 3.2 years *vs* 59.7 ± 7.9 years, *p*=0.004) than those in NDM-pD group (**Table 3**). When comparing within diabetic groups, FPG (137.7 ± 30.2 mg/dL *vs* 139.2 ± 23.49, *p*=0.859) and HbA1c (7.5 ± 1.1% *vs* 7.5 ± 0.86%, *p*=0.963) were comparable in both DM-pD and DM-iD groups. Serum pentosidine level was slightly higher in DM-pD in comparison to those in DM-iD group (7.3 ± 4.7 ng/mL *vs* 5.2 ± 2.53 ng/mL, *p*=0.120); however, the difference did not reach statistical significance (**Table 3**). Because FPG, HbA1c and serum pentosidine level were comparable between DM-pD and DM-iD, those factors should not influence the maintenance of osteogenic differentiation ability in cases of diabetes. There were also no differences in other parameters between DM-pD and DM-iD, including BMI, SBP, DBP, duration of being diabetic, the dosage of metformin, the usage rate of anti-hypertensive agents and statins, LDL-C and eGFR (**Table 3**). In contrast, age showed significant differences between DM-pD and DM-iD. Individuals in DM-iD were older than those in DM-pD group (60.5 ± 7.4 years *vs* 54.4 ± 3.2 years, *p*=0.001) but were at the same age as those in NDM-pD (60.5 ± 7.4 years *vs* 59.7 ± 7.9 years, *p*=0.701) (**Table 3**). The multivariate analysis demonstrated that age correlated with the potential for osteogenic differentiation in the PBMC-isolated from patients with diabetes, indicating that age is an independent risk factor for determining the differentiation potential toward osteoblasts of the PBMC-isolated from individuals with T2DM. Therefore, younger age was a protective factor for the preservation of osteoblast differentiation potential in T2DM.

**Table 3:** Factors determining osteogenic differentiation in type 2 diabetes.

Parameters	NDM-pD (n=27)	DM-pD (n=16)	*p*-value*	DM-iD (n=24)	*p*-value#
Age (years)	59.7 ± 7.9	54.4 ± 3.2	0.004	60.5 ± 7.4	0.001
BMI (kg/m²)	25.2 ± 3.2	25.7 ± 4.6	0.705	25.8 ± 4.1	0.953
FPG (mg/dL)	96.1 ± 9.4	137.7 ± 30.2	<0.0001	139.2 ± 23.4	0.859
HbA1c (%)	6.0 ± 0.4	7.5 ± 1.1	<0.0001	7.5 ± 0.8	0.963
LDL-C (mg/dL)	107.9 ± 28.9	96.1 ± 32.9	0.249	103.4 ± 48.1	0.601
DM duration (years)	–	5.1 ± 3.3	–	5.8 ± 4.6	0.615
Metformin dosage (mg/day)	–	1709.4 ± 655.3	–	1614.6 ± 619.3	0.646
Other drugs (% use)					
•ACEI or ARB** •DHP-CCB^##^ •Thiazide-like diuretics •Statins	46.2 50.0 15.4 70.3	75.0 50.0 18.8 81.3	0.109 1.000 1.000 0.494	62.5 62.5 20.8 78.2	0.503 0.522 1.000 1.000
Microvascular complications (%)	–	50.0		45.8	0.796
Macrovascular complications (%)	–	6.3		12.5	0.519
eGFR (ml/min)	86.3 ± 14.9	89.9 ± 12.6	0.442	82.6 ± 19.1	0.156
Pentosidine (ng/mL)	3.7 ± 1.8	7.3 ± 4.7	0.008	5.2 ± 2.5	0.120
sRAGE (pg/mL)	597.1 ± 422.4	519.1 ± 281.6	0.516	532.4 ± 232.2	0.871

*comparison between NDM-pD and DM-pD; #comparison between DM-pD and DM-iD.

**ACEI, angiotensin-converting enzyme inhibitors; **ARB, angiotensin II receptor blockers.

^##^DHP-CCB, dihydropyridine calcium channel blockers.

### *AGER* Overexpression Was Associated With Enhanced Cellular Apoptotic Signals and Impaired Osteogenic Differentiation, and Was Influenced by Age

Our previous study demonstrated higher *AGER* expression in patients with long-standing type 2 diabetes compared to that in non-diabetic controls, as well as the association of that RAGE overexpression with cellular apoptotic signal enhancement and osteogenic differentiation impairment. In this study, we analyzed PBMC isolated from 40 diabetic participants with metformin-monotherapy and 27 non-diabetic participants showing osteogenic differentiation (NDM-pD) to explore whether there was: 1) cellular RAGE overexpression in early-stage diabetes, 2) an association between RAGE overexpression with cellular apoptotic signal enhancement and osteogenic differentiation impairment, and 3) factors influencing cellular RAGE overexpression.

**Figure 2:**
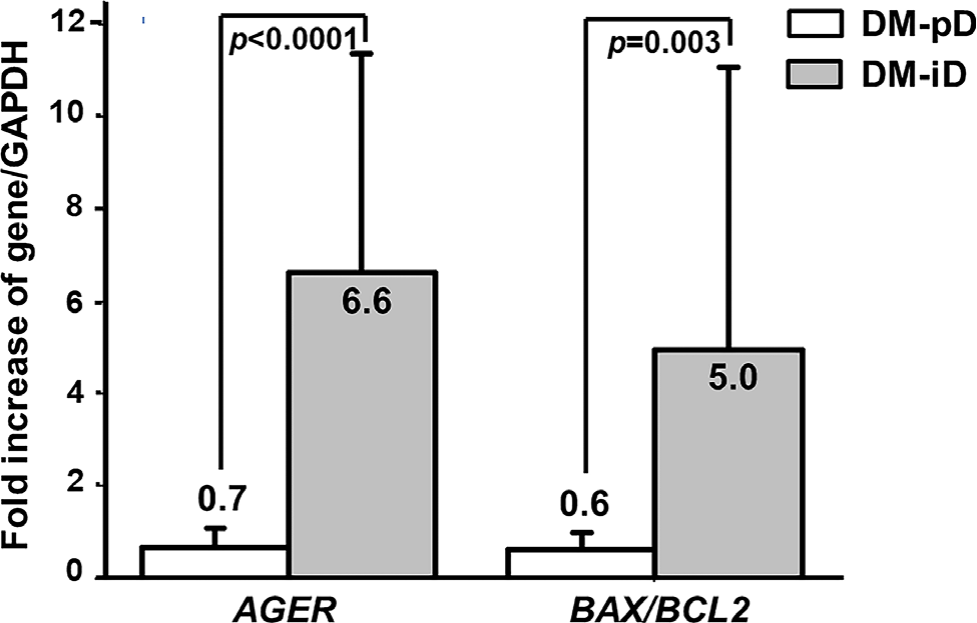
The expression of AGER, BAX and BCL2 genes. Comparison of the expression of the AGER, and BAX/BCL2 ratio between PBMC-isolated from subjects with diabetes with preserved osteogenic differentiation potential (DM-pD) and PBMC-isolated from diabetics with impaired osteogenic differentiation potential (DM-iD) by using age- and pentosidine-matched PBMC-isolated from non-diabetics with preserved osteogenic differentiation (NDM-pD) as a reference group. DM-iD showed higher expression of AGER and BAX/BCL2 ratio than the reference by 6.6 and 5.0 times, respectively, while DM-pD showed similar expression of AGER and BAX/BCL2 ratio to the reference. The expression of AGER and BAX/BCL2 ratio in DM-iD was significantly higher than that in DM-pD.

Since pentosidine has been documented in other studies as being an *AGER* enhancer, and age was confirmed earlier as an independent risk factor for the determination of osteogenic differentiation potential, the expression of *AGER* in both DM-iD and DM-pD was compared with the level of *AGER* expression in age- and pentosidine-matched NDM-pD individuals. This comparison aimed to explore if there was only a higher level of *AGER* expression in DM-iD, which would in turn suggest a link between cellular RAGE overexpression and defects in osteogenic differentiation. Using NDM-pD as a reference group, *AGER* expression in DM-iD was 6.6 times higher than that in the reference while the *AGER* expression in DM-pD was comparable to that in the reference (**Figure 2**). The *AGER* expression in DM-iD was significantly higher than that in DM-pD (6.6-fold *vs* 0.7-fold, *p*<0.0001), suggesting cellular RAGE overexpression in DM-iD as well as a connection between that RAGE overexpression and osteogenic differentiation defects in the PBMC. Consistent with higher *AGER* expression, the *BAX/BCL2* expression ratio in DM-iD was 5.0 times higher than that in the reference while the *BAX/BCL2* expression ratio in DM-pD was comparable to that in the reference (**Figure 2**). The *BAX/BCL2* expression ratio in DM-iD was also significantly higher than in DM-pD (5.0-fold *vs* 0.6-fold, *p*=0.003), suggesting higher cellular apoptotic rate only in cases of diabetes with impaired osteogenic differentiation ability. Furthermore, *AGER* expression showed a strongly positive correlation with the *BAX/BCL2* expression ratio (r=0.735, *p*<0.001), but showed a negative correlation with multiple osteoblast-specific gene expression including *ALPL* (r= -0.757, *p*<0.001), *BGLAP* (r= -0.670, *p*<0.001), *COL1A1* (r= -0.478, *p*=0.003) and *RUNX2/PPARγ* ratio (r= -0.770, *p*<0.001) (**Table 4**). These association analyses suggest a direct relationship between cellular RAGE overexpression and cellular apoptotic signals, as well as an inverse relationship between cellular RAGE overexpression and osteogenic differentiation ability in the PBMC derived from diabetic patients. Interestingly, *AGER* expression showed a positive correlation with age (r=0.470, *p*=0.003) (**Table 4**), suggesting that age influences cellular RAGE overexpression.

**Table 4:** Factors associated with *AGER* expression.

Parameter	*r*	*p*-value
**Demographic parameters**		
Age (years)	0.470	0.003
BMI (kg/m²)	0.042	0.807
DM duration (years)	0.052	0.769
**Serum markers**		
FPG (mg/dL)	-0.002	0.988
HbA1c (%)	-0.111	0.512
Pentosidine (ng/mL)	-0.039	0.819
sRAGE (pg/mL)	0.082	0.629
**Gene expression**		
* BAX/BCL2* ratio	0.735	<0.001
* ALPL*	-0.757	<0.0001
* BGALP*	-0.670	<0.0001
* COL1A1*	-0.478	0.003
* RUNX2/PPARγ* ratio	-0.770	<0.0001

## Discussion

This study demonstrated the preservation of osteogenic differentiation in 40% of PBMC-derived from type 2 diabetic patients who have had diabetes for an average of 5 years and are being treated with metformin monotherapy. Higher *AGER* expression was demonstrated only in PBMC-isolated from diabetics with poor osteogenic differentiation. This study not only demonstrated the existence of cellular RAGE overexpression in early stages of type 2 diabetes but also strengthened the link between that cellular RAGE overexpression and osteogenic differentiation retardation. This study also provided evidence to suggest that cellular RAGE overexpression increased with age. In addition, age and *AGER* expression were shown to be independent risk factors determining osteogenic differentiation potential of the PBMC-derived from T2DM.

In animal models with T2DM, a dramatic decrease in osteoblast/osteoid surface and bone mineral apposition rate has been demonstrated which indicates osteoblast differentiation and function impairment (46). Consistent with findings from animal studies, a reduction in osteoblast/osteoid surface, osteoid volume and thickness, which indicates a low bone formation state, has also been shown in humans with T2DM (10, 11, 47). Our previously study also demonstrated an impairment in osteogenic differentiation of the PBMC-isolated from long-standing type 2 diabetic patients (41). In agreement with previous studies, the present study illustrated impaired osteogenic differentiation in cases of type 2 diabetes. In the multivariate analysis, being diabetic significantly increased the risk of osteogenic differentiation impairment by 13.5 times (OR 13.5; 95% CI 3.21-77.91; *p*<0.001). In this study, 40% of cells from diabetic patients expressed osteoblast differentiation markers to a similar extent of those in non-diabetic individuals, suggesting a preserved potential of osteogenic differentiation only in some of the population among the whole diabetic group. Even though being diabetic increases the risk of osteogenic differentiation defects, diabetes at the different stages may have different degrees of that defect. Our previous study, which included long-standing type 2 diabetic subjects with an average of 10.7 ± 7.7 years of diagnosis, demonstrated that only 7.3% of PBMC-isolated from these diabetic patients expressed osteoblast-specific genes while 86.7% in non-diabetic controls expressed those genes (7.3% *vs* 86.7%, *p*<0.0001) (41). However, the present study was carried out with cells from patients at an earlier stage of diabetes with an average of 5.5 ± 4.1 years of diagnosis and showed that 40% of PBMC-isolated from diabetic subjects expressed osteoblast-specific genes while 90% in non-diabetic controls expressed those genes (40% *vs* 90%, *p*<0.0001). The present study showed a much higher number of PBMC with preserved osteogenic differentiation potential than those in the previous study which was done at a later stage of diabetes. However, it remains to be elucidated whether newly diagnosed type 2 diabetes still results in an impaired osteogenic differentiation.

Our previous study demonstrated cellular RAGE overexpression in type 2 diabetes which may lead to higher cellular apoptosis and poorer differentiation toward osteoblasts in T2DM. Because most PBMC-isolated from diabetic patients lose their ability to differentiate into osteoblasts, no firm conclusion can be made regarding the influence of cellular RAGE overexpression shown in type 2 diabetes on cellular apoptosis and the poorer differentiation found in type 2 diabetes. In this study, we focused on the earlier stage of diabetes and we demonstrated that only 60% (24/40) of the PBMC-isolated from diabetic patients lose their osteogenic differentiation potential (DM-iD). Therefore, we had the opportunity to explore whether there was only cellular RAGE overexpression in PBMC-isolated from diabetic patients showing impaired osteogenic differentiation. As pentosidine is an *AGER* enhancer and age is an independent risk factor for determining osteogenic differentiation as described above, *AGER* expression in an age- and pentosidine-matched NDM-pD group was used as a reference to compare the level of *AGER* expression between DM-iD and DM-pD for determining whether higher cellular *AGER* expression occurred only in the DM-iD group. Interestingly, higher *AGER* expression was demonstrated in DM-iD group but not in DM-pD, suggesting the existence of RAGE overexpression only in PBMC with poor osteogenic differentiation ability that isolated from diabetic patients. To further explore the association between cellular apoptosis and osteogenic differentiation, *BAX* and *BCL2* expression in DM-iD and DM-pD were compared by using *BAX/BCL2* expression in an age- and pentosidine-matched NDM-pD group as a reference. As seen with *AGER* expression, the ratio of *BAX/BCL2* expression was higher only in the DM-iD group, suggesting a link between cellular apoptotic signal enhancement and osteogenic differentiation impairment. Consistent with our previous study (41), the expression of *AGER* and *BAX/BCL2* ratio showed a strong correlation with each other (r=0.735, *p*<0.001). Therefore, the present study not only confirmed the existence of RAGE overexpression in PBMC with poor osteogenic differentiation potential but also strengthened the link between the cellular RAGE overexpression with osteogenic differentiation impairment and cellular apoptotic signal enhancement in the PBMC-isolated from T2DM patients.

Since the hyperglycemic state in diabetes accelerates the accumulation of AGEs in various tissues including those in the skeleton, accumulation of AGEs is one of the factors proposed as being responsible for the impairment in bone quality associated with diabetes and may be a useful predictor of fracture in diabetic individuals. Several studies have shown that the skeletal accumulation of AGEs alters bone mechanical properties, leading to fragility fractures (22–24). In addition, studies in primary culture osteoblasts and mesenchymal stem cells demonstrated that AGEs attenuated osteoblast differentiation and enhanced osteoblast apoptosis (25–29). In humans, serum pentosidine showed a positive correlation with fracture risk in T2DM (32–34). In contrast, esRAGE, an AGE neutralizer, showed a negative correlation with fracture risk in T2DM (34, 35). In this study, serum pentosidine was significantly higher in the group with diabetes than that in the age-matched non-diabetic group, implying accelerated accumulation of AGEs in diabetes mellitus. However, the levels of serum pentosidine in the group of diabetics with preserved osteogenic differentiation was comparable to the group of diabetics with impaired osteogenic differentiation. Moreover, a higher cellular *AGER* expression was illustrated only in the cells from the group of diabetics with impaired osteogenic differentiation, and that higher *AGER* expression was shown to be associated with the differentiation potential toward osteoblasts. Therefore, it is conceivable that serum pentosidine level has poor potential for the prediction of osteogenic differentiation in diabetes. Nevertheless, due to a small sample size and the wide standard deviation of serum pentosidine concentration found, the results of our study cannot exclude its potential for predicting osteoblast differentiation ability.

As 40% of PBMC-isolated from the diabetic group were shown to maintain their osteogenic differentiation potential, there was a potential for the exploration of the factors determining the preservation of osteogenic differentiation ability in our present study. These factors will be valuable in future exploration for guidance of fracture prevention in type 2 diabetes. Using the results of the multivariate analysis, this study demonstrated that age was a factor showing a correlation with the osteogenic differentiation potential of the PBMC-isolated from individuals with diabetes, indicating that age is an independent risk factor for determining the differentiation potential towards osteoblasts of the PBMC in diabetics. There is a higher probability for the preservation of the osteogenic differentiation potential in the younger diabetic individuals. In addition to age, *AGER* expression was also a risk factor for determining osteogenic differentiation, suggesting the contribution of higher cellular RAGE overexpression to the potential of differentiation of the PBMC towards osteoblasts. Interestingly, this study also demonstrated a positive correlation between age and *AGER* expression. Since age was an independent risk factor for osteogenic differentiation, as well as age showing a positive correlation with *AGER* expression, age itself is conceivably a contributor to cellular RAGE overexpression which in turn negatively affects osteogenic differentiation. Son and colleagues (48) demonstrated that accumulation of tissue AGEs and AGE-RAGE binding intensity increased with age and were different in organs in a non-diabetic mice model. In the liver and kidney, AGEs progressively accumulated with age, and AGE-RAGE binding intensity also increased with age, leading to an increase in its downstream signaling cascade. To the contrary, the accumulation of AGEs and AGE-RAGE binding intensity did not increase with age in skeletal muscles. Therefore, it remains a need for further elucidation as to whether RAGE activation in the skeleton conforms to an age-dependent pattern, leading to the probability of higher RAGE activation with increasing age. If AGEs-RAGE activation in the skeleton is an age-dependent pattern, being diabetic would drive accelerated accumulation of AGEs and even perpetuate RAGE activation as individuals inevitable grow older, resulting in the accelerated impairment of bone quality and increased fragility fracture in type 2 diabetes. From the evidence shown in this study, being diabetic increased the risk of osteogenic differentiation impairment and younger age is the single protective factor identified for preservation of osteogenic differentiation in T2DM, it is pertinent to state that prevention of becoming diabetic may be the most effective way to preserve the potential for osteogenic differentiation of the PBMC.

Several different types of medication prescribed for participants in this study were shown to influence bone metabolism. Metformin was the only anti-hyperglycemia agent given to all diabetic individuals in this study and it was not taken by non-diabetic controls. Metformin has been shown to promote the differentiation of osteoblasts from mesenchymal stem cells through the activation of the AMPK pathway (49, 50). Zhou Z and colleague (51) also demonstrated that metformin suppresses AGE-dependent RAGE activation in bone marrow derived macrophages. Even though all diabetic participants in this study may get benefit from metformin therapy, this medication did not overcome the detrimental effects of diabetes on osteogenic differentiation in 60% of cases. Metformin was used at the same dosage in both DM-pD and DM-iD, suggesting that the quantity of metformin did not influence the preservation of osteogenic differentiation ability found in this study. ACEI and ARB have been previously shown to have beneficial effects on bone metabolism and fractures (52–54). Liu YY and colleagues (52) showed that captopril, a type of ACEI, promoted osteoblast differentiation of primary cultured osteoblast cells as well as enhanced bone formation and bone strength in ovariectomized rats. In human, Kao YT and colleagues (53) showed that hypertensive individuals treated with ACEI or ARB had a decreased risk of osteoporotic fracture compared to those treated with other medications. Even though the difference did not reach statistical significance, participants in diabetic group used ACEI or ARB at a higher rate than those in non-diabetic group in this study (67.5% *vs* 44.8%, *p*=0.084). Therefore, it is reasonable to state that ACEI and ARB do not contribute to a lower rate of osteogenic differentiation in the diabetic group. In patients with diabetes, ACEI and ARB were used in the DM-pD group at a higher rate than those in DM-iD (75% *vs* 62.5%, *p*=0.503); however, the difference was nowhere near statistical significance. Therefore, it is reasonable to state that ACEI or ARB usage is not a contributory factor in the maintenance of osteogenic differentiation shown in DM-pD. Statins have been previously shown to have beneficial effects on bone metabolism and osteoporosis (55–58). Statins have been demonstrated to promote osteoblast differentiation and bone formation by stimulating the Akt/PI3 kinase and the β-catenin/Wnt signaling pathway, as well as to inhibit osteoblast apoptosis *via* activation of the TGFβ/Smad3 pathway (55). Zhang M and colleagues (56) demonstrated that simvastatin promoted differentiation of rat mesenchymal stem cells toward osteoblasts through the up-regulation of β-catenin (56). In humans, Lin TK and colleagues (57) carried out a nation-wide population cohort study to illustrate that statin use was associated with the decreased risk of osteoporosis in both females and males. In this study, participants in the diabetic group used statins at a higher rate than those in non-diabetic controls (79.5% *vs* 66.7%, *p*=0.275); however, the difference did not reach statistical significance. Therefore, from these findings the higher usage rate of statins in the diabetic group should not contribute to a lower rate of osteogenic differentiation in diabetic group.

This study provides evidence to support that cellular RAGE overexpression leads to osteogenic differentiation impairment in early stages type 2 diabetes, and that cellular RAGE overexpression is influenced by age. However, this evidence should be carefully interpreted due to several limitations. First, this study only demonstrated signal activation by mRNA level not protein expression due to the limited number of isolated cells from the relatively small 35-40 mL sample of peripheral blood collected from recruited patients. This raises the possibility the chance that transcription is activated but not translated into proteins. Therefore, it is probable that *AGER* overexpression will not lead to higher RAGE activation. The further studies involving RAGE knock-down would clarify whether cellular RAGE overexpression directly entail an impaired osteoblast differentiation. Second, this study presented data to show the pattern of association between parameters, so the causes and effects of those parameters cannot be definitely concluded. Finally, this study was a cross-sectional study which had several unexpected confounding factors by the nature of this type of study, for examples, *AGER* polymorphisms. Even though all baseline characters of the enrolled participants were generally comparable, those unexpected confounding factors might influence the results of the study. Multiple single nucleotide polymorphisms (SNPs) of *AGER* gene have been reported for association with diabetes and chronic diabetic complications (58–60). Cheng H and colleagues (58) showed that rs1800624 and rs2070600 SNPs associated with an increased risk of type 2 diabetes in South Asians and Caucasian, respectively. The rs1800624 SNP was documented for increasing *AGER* expression *in vitro* by enhancing the binding affinity of the transcription factor site while the rs207600 SNP was documented for enhancing the affinity of RAGE for its ligands (59). Since *AGER* polymorphism was not determined in this study, the contribution of *AGER* polymorphism to RAGE overexpression in PBMC was still possible. However, the evidence showing detrimental effects of *AGER* polymorphism on bone metabolism remains to be elucidated in cases of diabetes. To date, Raska Jr I and colleagues (60) showed that both rs1800624 and rs2070600 did not associate with sRAGE level, bone mineral density and fractures in postmenopausal women with T2DM.

## Data Availability Statement

The original contributions presented in the study are included in the article/supplementary material. Further inquiries can be directed to the corresponding author.

## Ethics Statement

The studies involving human participants were reviewed and approved by the Research Ethics Committee of the Faculty of Medicine, Chiang Mai University. The patients/participants provided their written informed consent to participate in this study.

## Author Contributions

MP was involved in conceptualization, funding acquisition, methodology, formal analysis, original draft writing, review and editing of the manuscript. PP was involved in the methodology, original draft writing, review and editing of the manuscript. PK was involved in original draft writing, as well as review and editing of the manuscript. All authors contributed to the article and approved the submitted version.

## Funding

This work is supported by Merck. The funder had no roles in the study design, data collection and analysis, preparation of the manuscript or decision to publish.

## Conflict of Interest

The authors declare that the research was conducted in the absence of any commercial or financial relationships that could be construed as a potential conflict of interest.

## Publisher’s Note

All claims expressed in this article are solely those of the authors and do not necessarily represent those of their affiliated organizations, or those of the publisher, the editors and the reviewers. Any product that may be evaluated in this article, or claim that may be made by its manufacturer, is not guaranteed or endorsed by the publisher.
